# Clinically Occult Pituitary Adenoma Can Appear as a Hypermetabolic Lesion on Whole Body FDG PET Imaging in a Patient with Lymphoma

**DOI:** 10.4274/Mirt.258

**Published:** 2013-04-05

**Authors:** İnanç Karapolat, Güray Öncel, Kamil Kumanlıoğlu

**Affiliations:** 1 Sifa University, Nuclear Medicine, Izmir, Turkey; 2 Sifa University, Radiology , Izmir, Turkey; 3 Ege University, Nuclear Medicine, Izmir, Turkey

**Keywords:** Fluorodeoxyglucose F18, pituitary adenoma, Positron-emission tomography

## Abstract

We report a case with Non-Hodgkin Lymphoma with a focus of intense hypermetabolism in the sellar region in the primary staging and posttreatment whole body F-18 FDG PET. Further evaluation with magnetic resonance imaging after posttreatment FDG PET revealed a pituitary adenoma. Endocrinologic workup was normal consistent with nonfunctioning pituitary adenoma and endocrinologists decided to follow up the patient by yearly magnetic resonance imaging. This case demonstrates a nonfunctioning pituitary adenoma by whole body FDG PET and emphasizes the importance of pursuing incidental findings detected in the sella on PET imaging.

**Conflict of interest:**None declared.

## INTRODUCTION

Pituitary adenoma is a relatively common tumor, originating from the pituitary gland (1). It can be classified by size and hormone secretion status. The majority of pituitary incidentalomas are microadenoma. Pituitary glands do not normally accumulate fluorine 18-fluoro-2-deoxy-glucose (FDG) and are not visualized on fluorine 18-fluoro-2-deoxy-glucose positron emission tomography (FDG PET) imaging (2,3,4). We describe a patient with Hodgkin’s Lymphoma (NHL) who was noted to have a focus of intense hypermetabolism in the sellar region in the primary staging and posttreatment whole body FDG PET which might be due to involvement of pituitary gland with lymphoma or a benign pituitary adenoma.

## CASE REPORT

**Figure 1:**
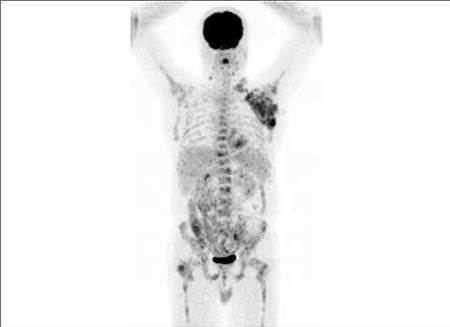
Pretreatment FDG PET MIP image : There are widespread malignantlymphadenopathy and involvement of bone-marrow

**Figure 2:**
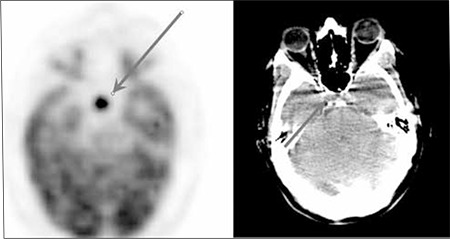
FDG PET and CT axial image : There is a focus of intense hypermetabolismin the sellar region which might be due to involvement ofpituitary gland with lymphoma or a benign pituitary adenoma

**Figure 3:**
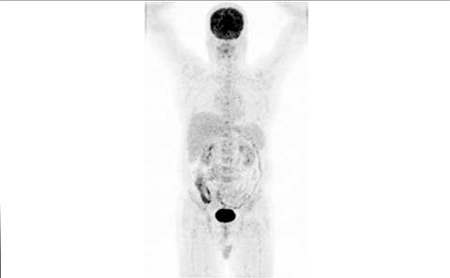
Posttreatment FDG PET MIP image : There are no hypermetabolicmalignant lymph nodes and bone marrow involvement consistent withcomplete response to therapy

**Figure 4:**
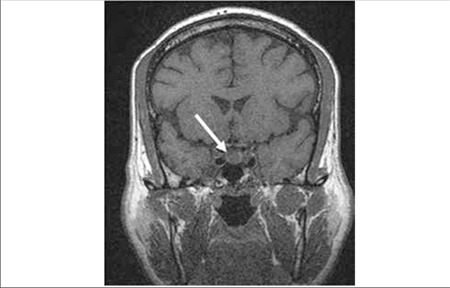
Precontrast T1W coronal image : The pituitary gland seems tobe mildly enlarged and there is a well-defined hypointense nodular lesionat the right side of the gland

**Figure 5:**
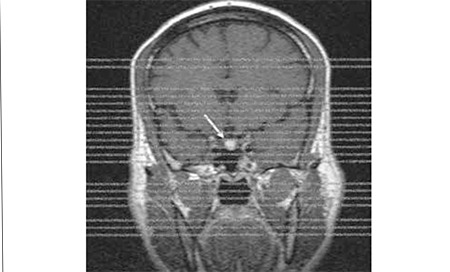
Postcontrast T1W coronal image : The nodular lesion showsdiffuse enhancement compared to the rest of the gland

A 49 year old man who presented with a mass located in the left axillary region enlarging during the last seven months. The patient underwent tru-cut biopsy and histopathological examination was consistent with non-Hodgkin’s lymphoma (small lymphocytic lymphoma). A whole body FDG PET was performed for staging which revealed widespread malignant lymphadenopathy and involvement of the bone-marrow (Figure 1). Also there was a focus of intense hypermetabolism (SUVmax=16.6) in the sellar region which might be due to malignancy or a benign pituitary adenoma (Figure 2). Further examination was recommended but the medical oncologist did not perform any other workup because patient had no clinical symptoms (headache, visual impairment, etc) and then the patient received chemotherapy. After treatment a second whole body FDG PET/CT was performed for response to therapy which revealed no hypermetabolic malignant lymph nodes and bone marrow involvement consistent with complete response to therapy (Figure 3). But still there was a focus of intense hypermetabolism (SUVmax=18.6) in the sella turcica strongly due to benign pituitary adenoma because malignancy was excluded by response to therapy. After FDG PET a gadolinium-enhanced magnetic resonance imaging (MRI) of the brain was performed and there was a 7.5x5.5 mm well-defined lesion in the pituitary gland with lack of enhancement compared with the normal pituitary gland, characteristic of pituitary adenoma (Figures 4,5). Endocrinologic evaluation revealed no hormonal abnormality and endocrinologist decided to follow-up the patient with yearly MRI.

## LITERATURE REVIEW AND DISCUSSION

Pituitary adenoma is a relatively common tumor, originating from the pituitary gland (1). Pituitary incidentalomas are defined as asymptomatic lesions of the pituitary gland found on MRI or computed tomography (CT) scans of the head performed for various purposes. They can be classified by size and hormone secretion status. Pituitary incidentalomas <10 mm are classified as microadenomas and those >10 mm are macroadenomas. Overall, the majority of pituitary adenomas are benign masses which remain clinically silent and are associated with minimal morbidity and mortality. The prevalence of pituitary incidentalomas discovered on autopsy is approximately 10%, with the majority of these lesions being microadenomas. The prevalence of pituitary incidentalomas discovered on neuroimaging such as MRI or CT is approximately 3.7–20% (2).

FDG PET/CT is used in the evaluation of most types of malignancy, which has resulted in significant clinical impact on patient management. FDG PET/CT is more accurate than CT or other conventional imaging modalities for the diagnosis of previously unknown, recurrent or metastatic cancer foci, based in part on its whole-body imaging capabilities. PET scanning is based on the principle that metabolically active cells such as those in tumors are more likely than normal cells to take up the labeled glucose analogue, FDG, to become detectable. However, several benign or non-neoplastic conditions avidly accumulate FDG, causing ambiguity in interpretation of results. (3) Incidental foci of abnormal FDG uptake detected during whole-body PET/CT examinations may represent previously unknown malignant sites, physiological variants or benign lesions unrelated to cancer.

Pituitary glands do not normally accumulate FDG and are not visualized on FDG PET imaging (2,3,4). There have been only a few previously reported cases with unexpected pituitary FDG uptake, primarily as case reports (2,3,4,5). The incidence of unexpected FDG-avid pituitary lesions was 0.073%, which is much lower than the incidence of FDG-avid incidentalomas in other endocrine organs such as the thyroid and adrenal glands (2). The majority of pituitary incidentalomas are microadenomas as in our case. Macroadenomas had higher FDG uptake than microadenomas. There have been no reports on the mechanism of FDG uptake in functioning and non-functioning pituitary adenomas. However, the size of a pituitary mass would be considered one of the important factors for the degree of pituitary FDG uptake.

We describe a patient with NHL who was noted to have a focus of intense hypermetabolism in the sellar region in the primary staging and posttreatment whole body FDG PET which might be due to involvement of pituitary gland with lymphoma or a benign pituitary adenoma. Campeau et al. reported on a patient with mucosa-associated lymphoid tissue lymphoma who had a non-functioning pituitary adenoma with FDG avidity (4). In our present case medical oncologist did not perform any further examinations because the patient had no clinical symptoms (headache, visual impairment, etc) and then the patient received chemotherapy. After treatment, a second whole body FDG PET/CT was performed for response to therapy which demonstrated persistent pituitary FDG uptake and disappearance of all hypermetabolic malignant foci. By contrast, Soussan et al. reported that pituitary gland involvement of NHL was detected on FDG PET/CT, and follow-up PET/CT after chemotherapy demonstrated total disappearance of focal pituitary FDG uptake (6). Therefore, we can postulate that the persistent pituitary FDG uptake seen in our patient could reflect benign etiology rather than metastasis.

In conclusion incidental pituitary uptake on FDG PET is a very rare finding. Most cases of incidental pituitary FDG uptake were diagnosed as clinically non-functioning adenomas, and there were a few functioning adenomas. Further evaluations, including hormone assays and pituitary MRI are warranted when pituitary uptake is found on FDG PET/CT and it is strongly due to benign etiology when persistent pituitary uptake seen on both pre and posttreatment PET scans.
